# Inhibition of autophagy enhances the cytotoxic effect of PA-MSHA in breast cancer

**DOI:** 10.1186/1471-2407-14-273

**Published:** 2014-04-21

**Authors:** Wen-Huan Xu, Zhe-Bin Liu, Yi-Feng Hou, Qi Hong, Da-Li Hu, Zhi-Ming Shao

**Affiliations:** 1 Department of Breast Surgery, Fudan University Shanghai Cancer Center, Shanghai 200032, P.R. China; 2 Cancer Institute, Fudan University Shanghai Cancer Center, Shanghai 200032, P.R. China; 3 Department of Oncology, Shanghai Medical College, Fudan University, Shanghai 200032, P.R. China; 4 Department of Oncology, Wuxi No. 4 People’s Hospital, Affiliated Hospital of Jiangnan University, 214062 Wuxi, Jiangsu, P.R. China; 5 Research and medical Department, Beijing Wanter Bio-pharmaceutical Co., Ltd, Beijing Huairou Yanqi Economic Technical Department Area, Beijing 101407, P.R. China

**Keywords:** PA-MSHA, ER stress, Autophagy, IRE1, Breast cancer

## Abstract

**Background:**

PA-MSHA, a genetically engineered Pseudomonas aeruginosa (PA) strain, is currently under investigation as a new anti-cancer drug. It can induce cell cycle arrest and apoptosis in different human cancer cells, including hormone receptor negative breast cancer cells. However, the underlying mechanism of tumor lethality mediated by PA-MSHA remains to be fully investigated.

**Methods:**

The effect of PA-MSHA on human hormone receptor negative breast cancer cells was analyzed by morphological measurement, western blot, cell proliferation assay and mouse xenograft model.

**Results:**

PA-MSHA was found to induce endoplasmic reticulum (ER) stress in breast cancer cell lines through the IRE1 signaling pathway. Inhibiting autophagy potentiated the cytotoxic effect of PA-MSHA while treating breast cancer cell lines. In mouse xenograft model, PA-MSHA produced more pronounced tumor suppression in mice inoculated with IRE1 gene knockdown. MDA-MB-231HM cells.

**Conclusions:**

These findings demonstrated inhibiting autophagy together with PA-MSHA might be a promising therapeutic strategy in treating hormone receptor negative breast cancer cells.

## Background

Breast cancer, one of the leading causes of cancer related mortality in women, is a disease with heterogeneous nature. Meanwhile “basal-like” breast cancer, ER and PR negative, is characterized by its aggressive behavior, distinct patterns of metastasis and lack of targeted therapies [1,2]. PA-MSHA, a genetically engineered Pseudomonas aeruginosa strain, has been successfully used as a protective vaccine [3] for adjuvant therapy of lymphoma and lung cancer. In recent preclinical studies, cytotoxic effect of PA-MSHA was observed in ER, PR negative breast cancer cells but not in ER, PR positive breast cancer cells [4]. The same effect was also exhibited in human hepatocarcinoma cells treated by PA-MSHA [5]. Given the increasing prevalence of PA-MSHA usage on cancer patients, further laboratory investigation are needed to better understand its anticancer mechanism.

Many chemotherapeutic drugs induce cell death via the endoplasmic reticulum (ER) stress mediated apoptotic pathway [6,7]. ER is composed of membranous tubules and vesicles. It serves cells with a Ca^2+^ reservoir and facilitates the secretion of properly folded proteins [8,9]. Disturbances in normal ER process lead to accumulation of unfolded proteins and trigger the unfolded protein response (UPR), which compensate the damage by reducing global protein synthesis and elicit autophagy, an alternate degradation system [10-12]. IRE1 and PERK/eIF2α are reported to be involved in the induction of autophagy upon ER stress.Autophagy can prevent the accumulation of toxic components in cells by sequestering cytoplasmic materials to autophagic vesicles and degrading them in the lysosome and recycling these materials [13]. In many studies, autophagy was induced while cancer cells faced with therapeutic stress, such as chemotherapy, radiotherapy and endocrine therapy [14].

In present study, we found that autophagy was stimulated in breast cancer cells upon ER stress of PA-MSHA through IRE1 pathway. Inhibition of autophagy promoted apoptosis both in vivo and in vitro. Our results provide molecular evidence that inhibiting autophagy will enhance PA-MSHA induced apoptosis in HR negative breast cancers.

## Methods

### Cell lines and materials

Human breast cancer cell lines MDA-MB-231 and MDA-MB-468 were obtained from the American Type Culture Collection. MDA-MB-231HM cell line was established by subclone selection procedure in our institute. The MDA-MB-231HM cell line has a high potential to metastasize to the lung and its establishment has been described previously [15]. The PA-MSHA used in this study was same as we used in our previous study [4]. Following reagents and primary antibodies were used: anti-LC3 (Cell Signaling Technology, Danvers, MA), anti-GAPDH, anti-caspase3, anti-cleaved-caspase3, anti-CHOP, anti-IRE1-a, anti-ATG5 (Santa Cruz Biotechnology Inc., Santa Cruz, CA); 3-MA and tunicamycin (Sigma-Aldrich, St. Louis, MO, USA). Lipofectamine 2000 reagent was obtained from Invitrogen (Cat. No 11668-019).

### Western blot

Cell lysates were prepared by extracting proteins with lysis buffer. Proteins were separated by sodium dodecyl sulfate polyacrylamidel gel electrophoresis and transferred to PVDF membranes. The membranes were blocked and incubated with primary antibodies. After incubation with peroxidase-conjugated secondary antibodies, the blots were visualized by enhancing chemiluminescence reagents.

### Transmission electron microscopy

Transmission electron microscopy was used to determine the effect of PA-MSHA treatment on the ultrastructure of breast cancer cells as described by Watkins and Cullen [16]. Ultra thin sections (65 nm) were examined under a JEM-100CX transmission electron microscope (JEOL, Japan) at × 84,00 or × 15,000 magnification.

### Flow cytometry with annexin V-FITC and PI staining

Cells were pretreated with solution containing of 2 mM 3-MA, 10 × 10^8^ cells/ml PA-MSHA, or 3-MA in combination with PA-MSHA for 48 hours. Single-cell suspensions with at least 1 × 10^6^ cells/ml were made. Apoptotic analyses were done by flow cytometry (FCM) as previously described [17] using a FACScalibur system (Becton Dickinson Biosciences, San Diego, CA). Propidium iodide-negative and annexin V-positive cells were analyzed by quadrant statistics as apoptotic cells.

### Cell proliferation

Cytotoxic effect was evaluated by the Cell Counting Kit-8 (CCK-8; Dojindo Molecular Technologies Inc., Gaitherbury, MD) assay. Cells were treated with specified concentration of PA-MSHA, 3-MA or 3-MA in combination with PA-MSHA and incubated at 37°C for 12, 24, 36 and 48 hours. Then, 10 μl of CCK-8 was added to every well, and the cells were incubated for an additional 3 hours at 37°C, after which the absorbance at 450 nm was recorded using a 96-well plate reader (Sunrise Microplate Reader, Tecan US, Inc., Charlotte, NC).

### Lentiviral-mediated knockdown of IRE1

Short hairpin RNA molecules targeted against human IRE1 gene were designed and synthesized by Sangon Biotech., Shanghai, China. The sequences of shRNAs targeting IRE1 was 5’-CTACTGGATAAACTTGCTTCA-3’. Oligonucleotides were annealed and inserted into digested PLKO.1-puro. Production of the lentiviral particles were carried out according to the manufacturer’s protocol. MDA-MB-231HM and MDA-MB-231HM cells were infected with lentivirus particles containing the shRNA and stable transfectants were selected and cultured in medium containing 3 ng/μl puromycin. The PLKO.1 scramble plasmid was packaged as a negative control. The PLKO.1 puro plasmid, packaging plasmid, pCMV- dR8.91 and envelope VSV-G were purchased from Addgen (Cambridge, MA).

### Morphological measurement of apoptosis

The morphological changes of apoptosis were assayed under a fluorescence microscope following staining with Hoechst 33258. Cells were treated with specified concentration of PA-MSHA for 48 h at 37°C, and then stained with 5 mg/L Hoechst 33258 (Sigma, St. Louis, MO) for 30 min at 37°C, visualized under a fluorescence microscope with standard excitation filters. The apoptotic cells were visualized at × 400 magnification.

### Animal xenograft model

This study followed the ethical approval of Fudan University Experimental Animal Department for research involving animals. 4-6 weeks old female BALB/c nude mice used in the study were provided by Shanghai Institute of Material Medica, Chinese Academy of Science. 2 × 10^6^ cells/ml MDA-MB-231HM-shCON cells and MDA-MB-231HM-shIRE1 cells suspended in 0.1 ml PBS were implanted into the mammary fat pad of mice. A total of 24 mice were randomized and assigned into four groups in the study. These mice were given 0.1 ml PA-MSHA (2.2 × 10^10^ cells/ml) s.c treatment every other day. Tumor volume was measured twice per week with calipers and calculated using the formula V (mm^3^) = 0.52 × ab^2^ (a = length, b = width). Body weight was recorded twice a week. The mice were killed and autopsied 6 weeks after tumor inoculation. Tumors were dissected and snap frozen for molecular biology analyses.

### Statistical analysis

Statistical analysis was performed using the software of Statistical Package for the Social Sciences (SPSS) Version 15 for Windows (SPSS Inc., Chicago, IL). Student’s t tests were used to determine statistical significance of differences between experimental groups. A P-value of less than 0.05 was considered significant. Graphs were created with Excel software (Microsoft Office for Windows 2003).

## Results

### PA-MSHA induces ER stress in breast cancer cells

**Figure 1:**
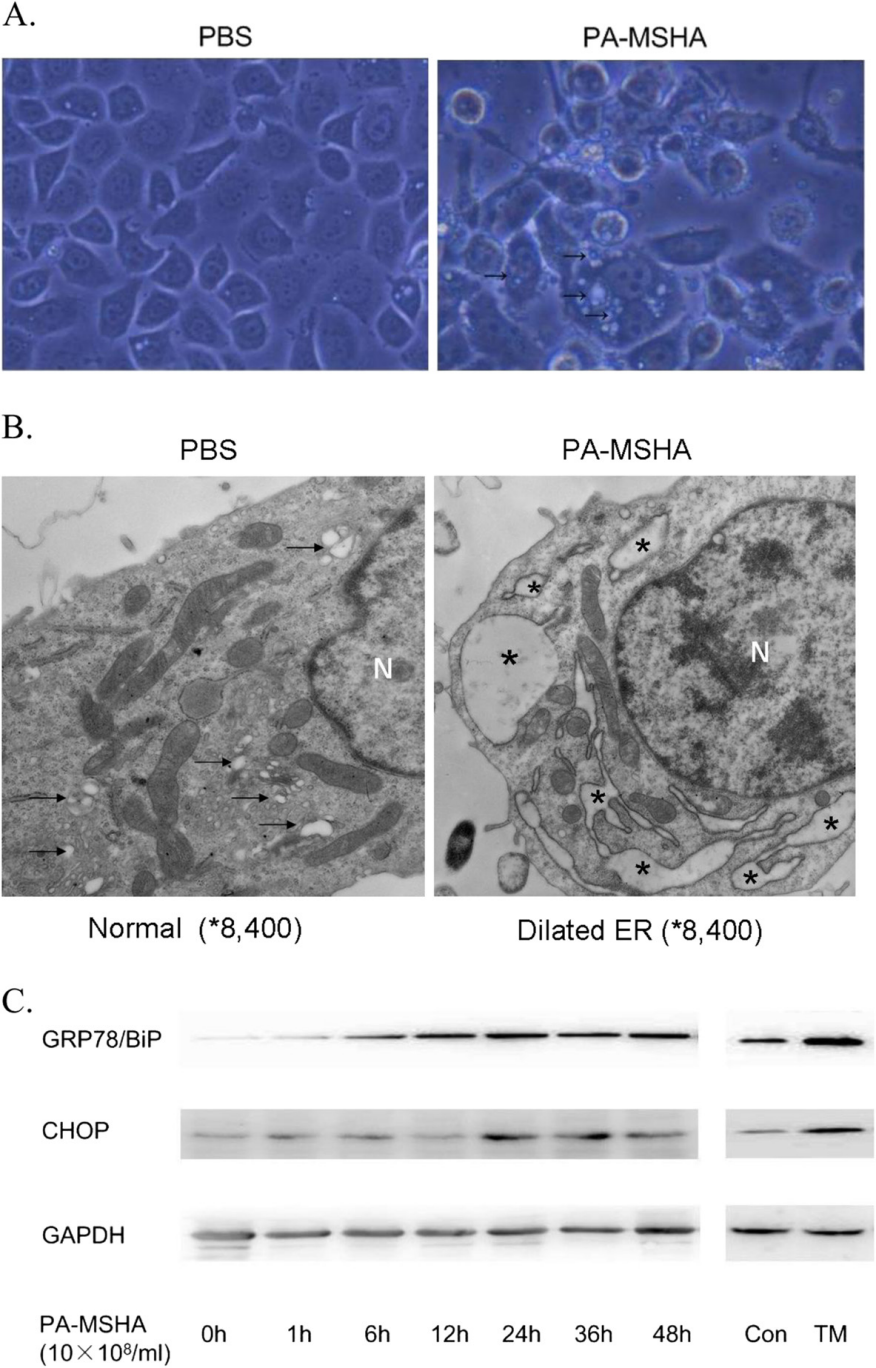
**ER stress induced by PA**-**MSHA in breast cancer cell lines. A**. MDA-MB-231HM cells treated by PBS or PA-MSHA (10 × 10^8^/ml) for 48 h were visualized by light microscopy. Increased cellular vacuolization was observed in PA-MSHA group. **B**. MDA-MB-231HM cells treated by PBS or PA-MSHA (10 × 10^8^/ml) for 48 h were visualized by electron microscopy (×8400). Cytoplasmic vacuoles were observed in PA-MSHA group (Figure1B). → point to normal ER. * indicate dilated ER cavity. N, Nucleus. **C**. The expression of GRP78/Bip and CHOP in MDA-MB-231HM cells exposed to PA-MSHA (10 × 10^8^/ml) at indicated time points. TM group: MDA-MB-231HM cells were treated by tunicamycin (5 mg/mL) for 24 hours. CON group: MDA-MB-231HM cells were treated by PBS for 24 hours.

Compared with cells treated with PBS, increased cellular vacuolization was observed in MDA-MB-231HM cells treated with PA-MSHA (10 × 10^8^ cells/ml) for 48 hours using light microscopy (Figure 1A). Further investigation with transmission electron microscopy identified dilated cytoplasmic vacuoles, which were previously described as indication of enhanced ER stress level [11], in these cells (Figure 1B). To verify the possibility that PA-MSHA might induce ER stress in breast cancer cells, we analyzed the expression levels of UPR targets GRP78/Bip and CHOP. Tunicamycin, a conventional ER stress agent, was used as a positive control. After 48 hours’ incubation with tunicamycin, elevated expression of GRP78/Bip as well as CHOP were detected in MDA-MB-231HM cells. Meanwhile significantly increased GRP78/Bip and CHOP were also found in cells incubated with PA-MSHA, as early as one hour after the treatment (Figure 1C). All these indicated that PA-MSHA can induce ER stress in MDA-MB-231HM cells.

### Autophagosome formation is activated upon PA-MSHA induced ER stress

**Figure 2:**
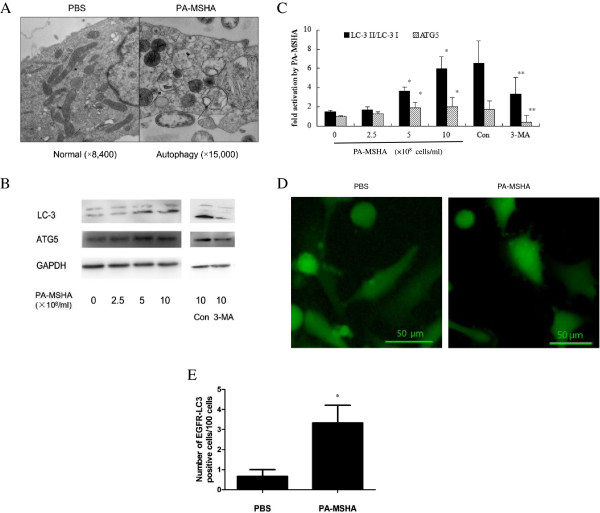
**Autophagy induced by PA**-**MSHA in breast cancer cell lines. A**. MDA-MB-231HM cells treated by PA-MSHA (10 × 10^8^/ml) for 48 h were visualized by electron microscopy (×8400). → point to autophagosome. * indicate dilated ER cavity. **B**. The expression of LC3 and ATG5 in MDA-MB-231HM cells treated by different dosage of PA-MSHA (0, 2.5, 5, 10 × 10^8^/ml). Con group: cells were treated by PA-MSHA (10 × 10^8^/ml) + PBS. 3MA group: cells were treated by PA-MSHA (10 × 10^8^/ml) + 3-MA (2 mM). **C**. The relative expression of LC3-II/LC3-I and ATG5 in MDA-MB-231HM cells treated by different dosage of PA-MSHA (2.5, 5, 10 × 10^8^/ml) to PA-MSHA (0 × 10^8^/ml) group and Con group. The values were the means +/- standard deviations of three independent experiments. * indicates a significant difference compared with PA-MSHA (0 × 10^8^/ml) group (*, P <0.05) . ** indicates a significant difference compared with Con group (**, P < 0.05). **D**. Punctate fluorescent patterns of EGFP-LC3 observed by fluorescence microscope in MDA-MB-231HM cells treated with PA-MSHA (10 × 10^8^/ml) for 24 hours. **E**. The percentage of EGFP-LC3 dots positive cells to the overall MDA-MB-231HM cells. Cells were treated with PA-MSHA (10 × 10^8^/ml) for 24 hours. The values were the means +/- standard deviations of three independent experiments. Asterisks indicate a significant difference from control cells (*, P <0.05) .

With electron microscopy, we noticed the co-existence of double-membrane vacuolar structures and dilated ER lumens in PA-MASH-treated breast cancer cells (Figure 2A). The double-membrane vacuolar structure was previously described as morphological feature of autophagosome. To testify the possibility that PA-MSHA can induce autophagy in breast cancer cells, 3MA, a conventional autophagy agent, was used as a positive control. The ratio of LC3-II/LC3-I and the expression of Atg5 increased as the dose of PA-MSHA rose. In cells treated with PA-MSHA as well as 3-MA, decreased ratio of LC3-II/LC3-I and expression of Atg5 was found (Figure 2B, C).

Characteristic punctate fluorescent patterns of EGFP-LC3 in cells treated with PA-MSHA for 24 hours were also observed, indicating the existence of autophagosome [18] (Figure 2D). Morphometric analysis of the EGFP fluorescence images revealed that the percentage of EGFP-LC3-punctate staining cells was 0.67% and 3.33% of the total cells in the absence and presence of PA-MSHA respectively. The EGFP fluorescence area increased about 4.97-fold after the treatment of PA-MSHA (Figure 2E). These indicated autophagy was activated by PA-MSHA.

### The IRE1 signaling pathway is required for activation of PA-MSHA-induced autophagy in breast cancer cells

ER stress was reported to trigger autophagy while facing cell damage stress in many studies [10-12]. IRE1 was involved in the induction of autophagy upon ER stress. Previously, we found elevated expression of CHOP in PA-MSHA treated breast cancer cells. Since CHOP was also reported to be up-regulated by IRE1, we postulated that IRE1 signaling pathway might be required for activation of PA-MSHA-induced autophagy in breast cancer cells. IRE1-shRNA was used to confirm our hypothesis.

**Figure 3:**
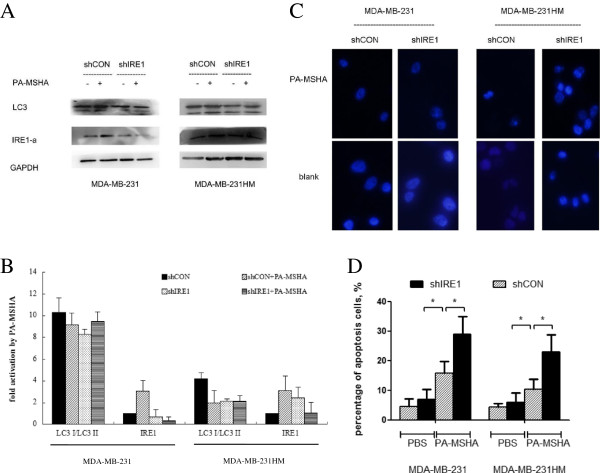
**Autophagy in breast cancer cells treated by PA**-**MSHA was induced by ER stress via IRE1 pathway. A** &**B**. The expression of LC3 and IRE1-a in MDA-MB-231-shIRE1, MDA-MB-231HM-shIRE1 or cells transfected with shCON vectors treated by PA-MSHA (10 × 10^8^ cells/ml)/PBS for 48 hours **(A)**. The relative expression of LC3-I/LC3-II and IRE1-a of each sample **(B)**. **C**. Nuclear staining of MDA-MB-231-shIRE1, MDA-MB-231HM-shIRE1 cells and cells transfected with shCON vectors with Hoechst 33258 (original magnification, ×400). Cells were treated with PA-MSHA (10 × 10^8^cells/ml) or PBS for 24 hours before examination. **D**. The percentage of apoptotic cells in MDA-MB-231-shIRE1, MDA-MB-231HM-shIRE1 cells was measured by flow cytometric analysis with Annexin V-FITC/PI staining. Cells were transfected with shCON vectors or shIRE1 vectors.

While treated with PA-MSHA, the LC3-II accumulation was decreased in cells transfected with shIRE1 compared with those transfected with shCON (Figure 3A, B). We also observed morphological changes in cell nuclei using Hoechst-33258. As shown in Figure 3C, dense and thin crowns of nuclear coloration, typical of chromatin condensation, were observed in MDA-MB-231HM-shIRE1 cells, and less typical morphological changes were observed in MDA-MB-231HM cells. Same changes were observed in MDA-MB-231-shIRE1 cells. Besides, using flow cytometric analysis, we found apoptosis induced by PA-MSHA was augmented in cells transfected with shIRE1 compared with those transfected with shCON (Figure 3D). All these results indicated that IRE1 pathway was required for activation of PA-MSHA-induced autophagy in breast cancer cells.

### Protective effects of autophagy during PA-MSHA-induced ER stress

**Figure 4:**
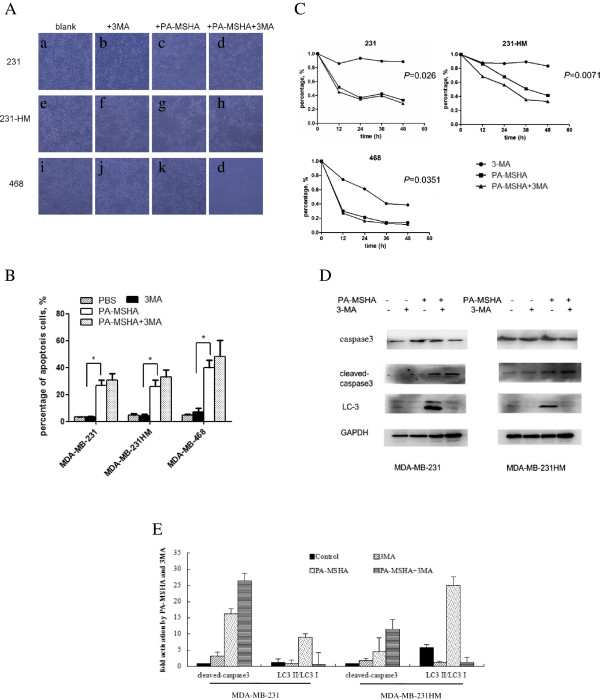
**Protective effects of autophagy. A**. Cells treated by 3-MA (2 mM), PA-MSHA (10 × 10^8^/ml) or PA-MSHA together with 3-MA for 48 hours were visualized by light microscopy. Most cellular vacuolization and death were found in cells treated with PA-MSHA and 3-MA. **B**. The percentage of apoptotic cells in MDA-MB-231-shIRE1, MDA-MB-231HM-shIRE1 cells, MDA-MB-468 cells was measured by flow cytometric analysis. Cells were treated by 3-MA (2 mM), PA-MSHA (10 × 10^8^/ml) or PA-MSHA together with 3-MA for 48 hours (*P < 0.05). **C**. The viability of cells treated by PA-MSHA (10 × 10^8^ cells/ml), 3-MA (2 mM) or PA-MSHA together with 3-MA was evaluated by Cell Counting Kit-8 assay. **D** &**E** The expression of caspase3, cleaved-caspase3 and LC3 in cells treated with PA-MSHA (10 × 10^8^ cells/ml), PBS, 3-MA (2 mM) or PA-MSHA together with 3-MA **(D)**. The relative expression of cleaved-caspase3 and LC3-II/LC3-I of each sample.

Using light microscopy, we found cellular vacuolization and death in MDA-MB-231 cells treated with PA-MSHA. Increased cellular vacuolization and death was observed in MDA-MB-231 cells treated with PA-MSHA and autophagy inhibitor 3-MA [19]. Similar results were found in MDA-MB-231HM and MDA-MB-468 cells (Figure 4A). This phenomenon was confirmed by flow cytometric analysis. Compared with groups treated by PBS, 3-MA (2 mM) or PA-MSHA, more apoptotic cells were found in the group treated by 3-MA (2 mM) and PA-MSHA (Figure 4B). By cell viability test, we also detected more apoptosis in the group treated by PA-MSHA and 3MA (Figure 4C). These findings were consistent with results indicated in Figure 4D and E. Compared with cells treated by PA-MSHA and 3MA, the ratio of LC3-II to LC3-I increased in cells treated by PA-MSHA. Meanwhile apoptosis-associated caspase3 activity was activated in MDA-MB-231 cells treated with PA-MSHA in combination with 3-MA, indicating that inhibition of autophagy could sensitize breast tumor cells to the cytotoxic (apoptotic) actions and PA-MSHA-induced autophagy might be a cyto-protective mechanism in hormone negative breast cancer cells.

### Tumor suppression induced by PA-MSHA is enhanced by inhibiting autophagy

Previous study showed that inhibiting autophagy in vitro would result in more death in breast cancer cells treated with PA-MSHA. We next assessed whether suppression of autophagy would also potentiate the cytotoxic effects of PA-MSHA in vivo. We divided nude mice in four groups: (a) mice implanted with MDA-MB-231HM-shCON cells treated with vehicle only, (b) mice implanted with MDA-MB-231HM-shCON cells treated with PA-MSHA only, (c) mice implanted with MDA-MB-231HM-shIRE1 cells treated with vehicle only, (d) mice implanted with MDA-MB-231HM-shIRE1 cells and treated with PA-MSHA only. 6 weeks after inoculation, 5 out of 6 mice (83.3%) in the shIRE1 + PA-MSHA group were found with tumors. Rates of grafted tumor in the other 3 groups were 100%. One nude mouse in shCON + PBS group died the day before all the mice were killed.

**Figure 5:**
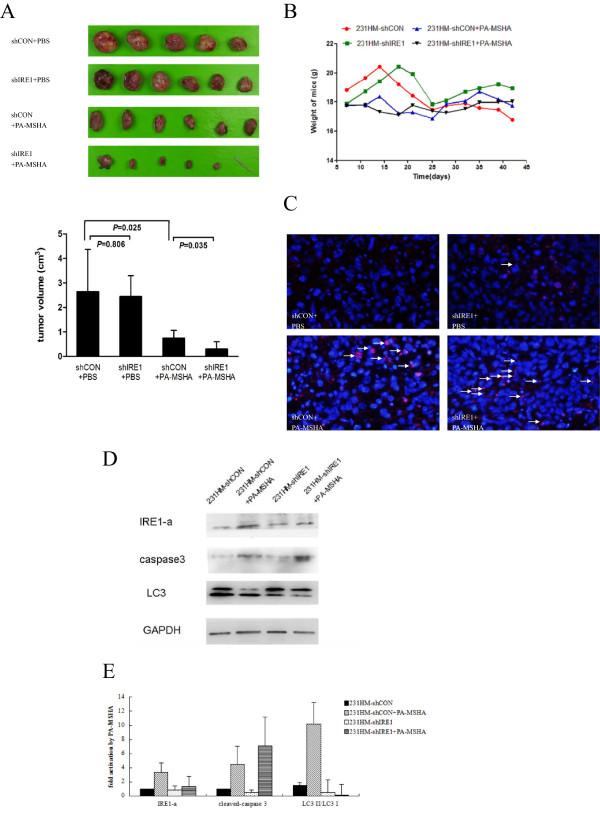
**Protective effect of PA**-**MSHA on tumor growth in vivo. A**. Tumor volume measured at the indicated time after MDA-MB-231HM -shIRE1, MDA-MB-231HM-shCON cells were implanted into the mammary fat pad of mice. *P < 0.01 **B**. Body weight of mice measured at the indicated time after MDA-MB- 231HM-shIRE1, MDA-MB-231HM-shCON cells were implanted into the mammary fat pad of mice. **C**. Apoptosis was measured by TUNEL staining of tumor tissue sections. Tissues were counterstained with DAPI to detect cell nuclei. **D** &**E**. The expression of IRE1-a, cleaved-caspase 3, LC3 in PA-MSHA-treated xenografts **(D)**. The relative expression of IRE1-a, cleaved-caspase3 and LC3-II/LC3-I of each sample.

As shown in Figure 5A, knocking down of IRE1 in MDA-MB-231HM cells did not reduce the growth rate of tumor xenografts (*P* = 0.806). PA-MSHA treatment led to significant tumor suppression in mice (*P* = 0.025). Furthermore, with the help of knocking down IRE1, PA-MSHA produced a greater decrease in tumor volume (*P* = 0.035). Meanwhile, treatment of PA-MSHA caused no significant loss in body weight in either MDA-MB-231HM-shCON or MDA-MB-231HM-shIRE1 inoculated mice (Figure 5B). More TUNEL-positive cells were found in tumors treated with PA-MSHA compared with tumors not treated with PA-MSHA. Largest number of TUNEL-positive cells were observed in PA-MSHA + shIRE1 combination group (Figure 5C). While treated with PA-MSHA, increased expression of cleaved-caspase3 and decreased ration of LC3-II/LC3-I was found in 231-HM-shIRE1 cells compared with 231-HM-shCON cells (Figure 5D, E).

## Discussion

PA-MSHA , successfully used as a vaccine [20], has recently been validated to induce cytotoxic effect against human carcinoma cells [5]. PA-MSHA can also inhibit the hormone receptor negative breast cancer cells in a mannose-sensitive manner [4]. However, the direct mechanism for tumor lethality mediated by PA-MSHA remains to be fully characterized.

In this study, we mainly focused on the tumor cytotoxic ability of PA-MSHA on the HR negative breast cancer cells. We found enlarged vacuoles in HR negative breast cancers upon treatment of PA-MSHA. One possible explanation for these vacuoles is the induction of the UPR. UPR is an adaptive process, it can block protein translation and allows cells to compensate for protein accumulation and misfolding in the ER. Elevated GRP78/Bip and CHOP expression, typical evidence of the activation of ER stress-dependent UPR signaling pathway, was also found in HR negative breast cancer cells treated with PA-MSHA.

However, if the damage is too severe and persistent, ER stress will trigger autophagy to avoid cell damage, which may include IRE1/CHOP or PERK/eIF2α pathway. In our study, elevated CHOP and autophagy following PA-MSHA treatment were observed. Knocking down of IRE1 decreased autophagy and enhanced cell death in cells upon PA-MSHA treatment. These results indicated autophagy, induced by IRE1, was a protective agent of ER stress.

In our study, more TUNEL-positive cells was found in PA-MSHA treated MDA-MB-231HM- shIRE1 cells inoculated tumors compared with MDA-MB-231HM- shCON cells inoculated tumors. Furthermore, increased apoptosis was observed after autophagy was compromised in vivo. This was consistent with previous reported studies. One study had demonstrated that autophagy induced by Epirubicin protected breast cancer cells [21]. Another study revealed that autophagy acted as a survival signal in CML cells treated with tyrosine kinase inhibitors (TKIs) and CML cells resistant to TKIs can be abrogated by autophagy inhibitors [22]. Endostatin was also reported to induce autophagy in addition to apoptosis in endothelial cells [23].

Since inhibiting autophagy can lead to cell death [24,25], it provides a novel strategy in cancer therapy. The data presented herein suggested that autophagy inhibitors might be useful alliance of PA-MSHA in future clinical trials. In our study, no significant body weight loss was found in shIRE1 inoculated mice. This indicated there might be no additional toxicity using shRNAs as autophagy inhibitors. It will also be interesting to investigate whether other known or novel pharmacological inhibitors of autophagy can enhance the anticancer activity of PA-MSHA. Chloroquine, which has been used safely for decades in patients which malaria prophylaxis, may be a good choice.

## Conclusions

To our knowledge, this is the first report showing that PA-MSHA can induce ER stress in hormone receptor negative breast cancer cell lines. The ER stress activated autophagy through IRE1 dependent pathway. Acting as a pro-survival mechanism, autophagy alleviated PA-MSHA induced ER stress and facilitated the development of PA-MSHA-acquired resistance. Our data suggested that blocking autophagy with either genetic or chemical inhibitors may enhance the cytotoxicity induced by PA-MSHA. The alliance of autophagy inhibitors and PA-MSHA might be considered in future clinical trials treating hormone receptor negative breast cancer patients.

## Abbreviations

CQ: Chloroquine; ER: Endoplasmic reticulum; IRE1: Inositol requiring enzyme 1; 3-MA: 3-methyladenosine; MSHA: Mannose-sensitive hemagglutinin; PA: Pseudomonas aeruginosa; UPR: Unfolded protein response.

## Competing interests

The authors declare that they have no competing interests.

## Authors’ contribution

W-HX and Z-BL contributed equally in the performance, analyzing and reporting of this article. Y-F Hou designed the study and analyzed data. QH and D-LH provided shIRE1 and PA-MSHA in the study. Z-MS designed the study. All authors read and approved the final manuscript.

## Pre-publication history

The pre-publication history for this paper can be accessed here:

http://www.biomedcentral.com/1471-2407/14/273/prepub
